# CuFeO_2_–NiFe_2_O_4_ hybrid electrode for lithium-ion batteries with ultra-stable electrochemical performance†

**DOI:** 10.1039/c9ra03187a

**Published:** 2019-09-02

**Authors:** Jun Young Cheong, Seokwon Lee, Jiyoung Lee, Haeseong Lim, Su-Ho Cho, Doh C. Lee, Il-Doo Kim

**Affiliations:** Department of Materials Science and Engineering, Korea Advanced Institute of Science and Technology 291 Daehak-ro, Yuseong-gu Daejeon 305-701 Republic of Korea idkim@kaist.ac.kr; Department of Chemical and Biomolecular Engineering, Korea Advanced Institute of Science and Technology 291 Daehak-ro, Yuseong-gu Daejeon 305-701 Republic of Korea

## Abstract

Stable electrode materials with guaranteed long-term cyclability are indispensable for advanced lithium-ion batteries. Recently, delafossite CuFeO_2_ has received considerable attention, due to its relative structural integrity and cycling stability. Nevertheless, the low conductivity of delafossite and its relatively low theoretical capacity prevent its use as feasible electrodes for next-generation batteries that require higher reversible capacities. In this work, we suggest a simple and straightforward approach to prepare CuFeO_2_–NiFe_2_O_4_ by introducing Ni precursor into Cu and Fe precursor to form NiFe_2_O_4_, which exhibits higher capacity but suffers from capacity fading, through sol–gel process and subsequent heat treatments. The presence of both NiFe_2_O_4_ and CuFeO_2_ is apparent, and the heterostructure arising from the formation of NiFe_2_O_4_ within CuFeO_2_ renders some synergistic effects between the two active materials. As a result, the CuFeO_2_–NiFe_2_O_4_ hybrid sample exhibits excellent cycling stability and improved rate capability, and can deliver stable electrochemical performance for 800 cycles at a current density of 5.0 A g^−1^. This work is an early report on introducing a foreign element into the sol–gel process to fabricate heterostructures as electrodes for batteries, which open up various research opportunities in the near future.

## Abstract 2

Novel NiFe_2_O_4_–CuFeO_2_ heterostructures were synthesized by sol–gel process and subsequent heat treatments, which exhibit excellent long-term high-rate cyclability.

## Graphical abstract

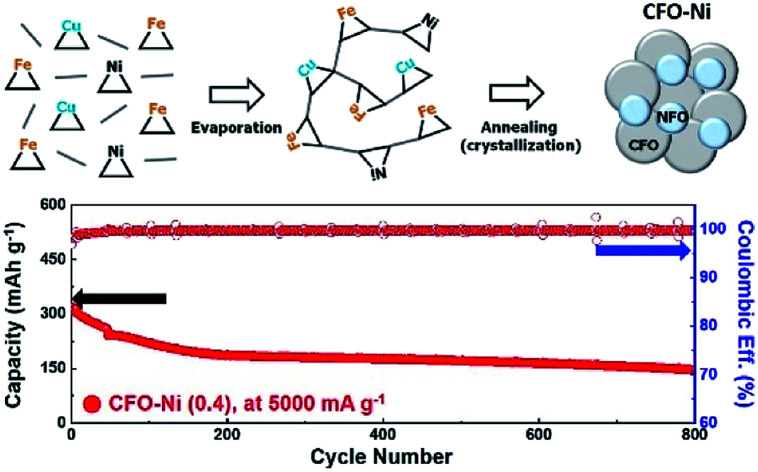

## Introduction

Lithium-ion batteries (LIBs), for the last three decades, have been researched and developed significantly among various kinds of energy storage systems.^1,2^ The applications of LIBs include electronic devices, electric vehicles (EV), and can also be extended to large scale public transportation and electric grids in near future.^3–5^ To realize next-generation LIBs, it is important to consider not only the high energy density but also the durability of the performance, where the given electrode material has a stable cycling upon high charge and discharge rates.^6–12^ In this regard, high rate cyclability and stable electrochemical performance are both key to the successful development of next-generation rechargeable batteries.

Recently, various Fe-based and metal oxide materials have been sought out as alternative anodes for secondary batteries.^13–17^ Among a number of candidates for electrode materials, delafossite materials have recently garnered much attention, as it is a ternary oxide that possesses a unique layered crystal structure.^18^ Such structure provides three-dimensional pathways, which are beneficial for facile ion insertion and de-insertion.^18^ Based on a number of previous reports,^18–20^ it has been demonstrated that CuFeO_2_ generally exhibits quasi-stable cycle retention characteristics. Nevertheless, the aggregation of particles (as well as pulverization) and the low conductivity of CuFeO_2_ have hampered its application as feasible electrodes for LIBs, which require more delicate material design.

So far, all approaches have been adopted to boost up the electrochemical performance of CuFeO_2_ by using carbonaceous materials, including combining with reduced graphene oxide^18^ and graphene.^19^ Although using carbon can significantly improve the electronic conductivity, it leads to the decreased loading amount of CuFeO_2_, which may limit the full utilization of CuFeO_2_. Moreover, some pre-steps were required to fabricate carbonaceous materials, which may take longer time and requires slightly more complicated process.

In this work, we have successfully fabricated predominantly CuFeO_2_–NiFe_2_O_4_ (delafossite–spinel) hybrid materials by simple sol–gel process with the addition of Ni precursor. By introducing Ni precursor into the sol–gel solution, Ni actively reacts with Fe to form a spinel structure (NiFe_2_O_4_), while the rest of the Cu atoms form additional delafossites (CuFeO_2_). The approach suggested in this work is very simple and one-step and is distinct from the previous works on CuFeO_2_ in that it not only retains loading amount of active materials but also improves the overall capacity, in terms of cycle retention and rate capabilities. For the final product, various kinds of CuFeO_2_–NiFe_2_O_4_ (denoted as CFO-Ni samples) were formed, which exhibit both the crystal structures of CuFeO_2_–NiFe_2_O_4_, with a slight formation of CuO, that may later act as a highly conductive electron pathway.^21^ Attributed to the rationally designed crystal structures of the electrodes, CFO-Ni exhibited excellent rate cyclability at a current density of 5000 mA g^−1^, which has yet been reported. The work presented here serves as milestone to easily fabricate various heterostructures using sol–gel process and apply them to rechargeable energy storage systems, which open up various opportunities in near future.

## Experimental

### Chemicals

Copper(ii) nitrate trihydrate (Cu(NO_3_)_2_·3H_2_O, Sigma Aldrich, 99.999%), iron(iii) nitrate nonahydrate (Fe(NO_3_)_3_·9H_2_O, Sigma Aldrich, 99%), nickel(ii) nitrate hexahydrate (Ni(NO_3_)_2_·6H_2_O, Sigma Aldrich, 99.999%), citric acid (C_6_H_8_O_7_, Sigma Aldrich, ≥99.5%), ethylene glycol (C_2_H_6_O_2_, Sigma Aldrich, anhydrous, 99.8%), and ethanol (C_2_H_5_OH, Sigma Aldrich, anhydrous, 99.5%) were used for the sol–gel synthesis.

### Synthesis of CuFeO_2_ and CuFeO_2_–NiFe_2_O_4_ composites

The synthesis of CuFeO_2_ (CFO) and CuFeO_2_–NiFe_2_O_4_ composites (CFO-Ni) was carried out by simple sol–gel process and subsequent heat treatment. Briefly, the synthesis of CFO was carried out as follows: Fe(NO_3_)_3_·9H_2_O (4 mmol), Cu(NO_3_)_2_·3H_2_O (4 mmol), and C_6_H_8_O_7_ (8 mmol) were mixed in 20 mL of ethanol. After stirring for 2 h, ethylene glycol (8.96 mmol) was added to the mixture, which was further stirred for 1 h. The resulting solution was evaporated at 120 °C overnight. The resulting powder was grinded, and then annealed in air at 450 °C for 5 h to remove all traces of organics. The resulting powder was then annealed under argon environment (200 mL min^−1^) at 700 °C for 12 h. For the synthesis of CFO-Ni heterostructures, the same procedure in the synthesis of CFO was used except that Ni(NO_3_)_2_·6H_2_O and additional Fe(NO_3_)_3_·9H_2_O were added into the CFO solution to carefully match the molar ratio between Ni, Cu, Fe, and O. Specifically, the amount of Ni(NO_3_)_2_·6H_2_O was determined by the desired molar ratio of Ni to Cu content. Notably, due to the facile formation of NiFe_2_O_4_ in the annealing steps, Fe deficiency can easily take place without an additional Fe content so that the formation of the CFO was suppressed. Thus, an additional Fe source was added as much as Ni could consume to form NiFe_2_O_4_.

### Cell assembly

The electrode material was initially slurry casted on Cu foil, together with the binder and conductive agent. Briefly, 80 wt% of active materials (CFO and CFO-Ni) were mixed together with 10 wt% of binder containing poly(acrylic acid)/sodium carboxymethyl cellulose (Sigma Aldrich, USA, wt%/wt% = 50/50), and 10 wt% of conductive agent (Super P carbon black) in the presence of water. The slurry casted Cu foil was initially dried at 50 °C for 10 min and later dried under vacuum at 150 °C for 2 h to allow suitable adhesion of binder to the active materials. The average mass loading of the active materials was about 2 mg cm^−2^. The electrochemical cells were assembled inside the glove box in Ar atmosphere. The slurry casted Cu foil is punched into circular shape (14ϕ) and assembled together with separator (Celgard 2325), counter electrode (Li metal foil), and liquid electrolyte (1.3 M lithium hexafluorophosphate (LiPF_6_) dissolved in the solvent mixture of ethylene carbonate/diethylene carbonate (EC/DEC) with 10 wt% of fluoroethylene carbonate (FEC)) (PANAX ETC.). Prior to the electrochemical cell testing, aging process was conducted for 24 h to allow the optimal electrochemical performance.

### Characterization

The morphological characteristics of CFO and CFO-Ni were examined by field emission scanning electron microscopy (FE-SEM, SU5000, Hitachi). To analyze the elemental composition, energy-dispersive X-ray (EDX) spectroscopy was conducted using FE-SEM (XL 30 S FEG, Philips, Netherlands) with a beam voltage of 10 kV. Both morphological and crystal properties of CFO and CFO-Ni were examined by high resolution transmission electron microscopy (HRTEM, Tecnai F20) operating at 200 kV. The crystal structures of CFO and CFO-Ni were confirmed by X-ray diffractometer (XRD, D/MX-200, Rigaku). The chemical states of both CuFeO_2_ and NiFe_2_O_4_ were analyzed by X-ray photoelectron spectroscopy (XPS, K-alpha, Thermo VG Scientific). To analyze the redox reactions with Li, cyclic voltammetry (CV) analysis was conducted in the scan rate of 0.1 mV s^−1^ using the battery testing device (Maccor Series 4000, KOREA THERMO-TECH). To further investigate the internal cell resistances, impedance tests were carried out using 1-channel potentiostat (ZIVE SP1, Wonatech).

## Results and discussion

**Fig. 1:**
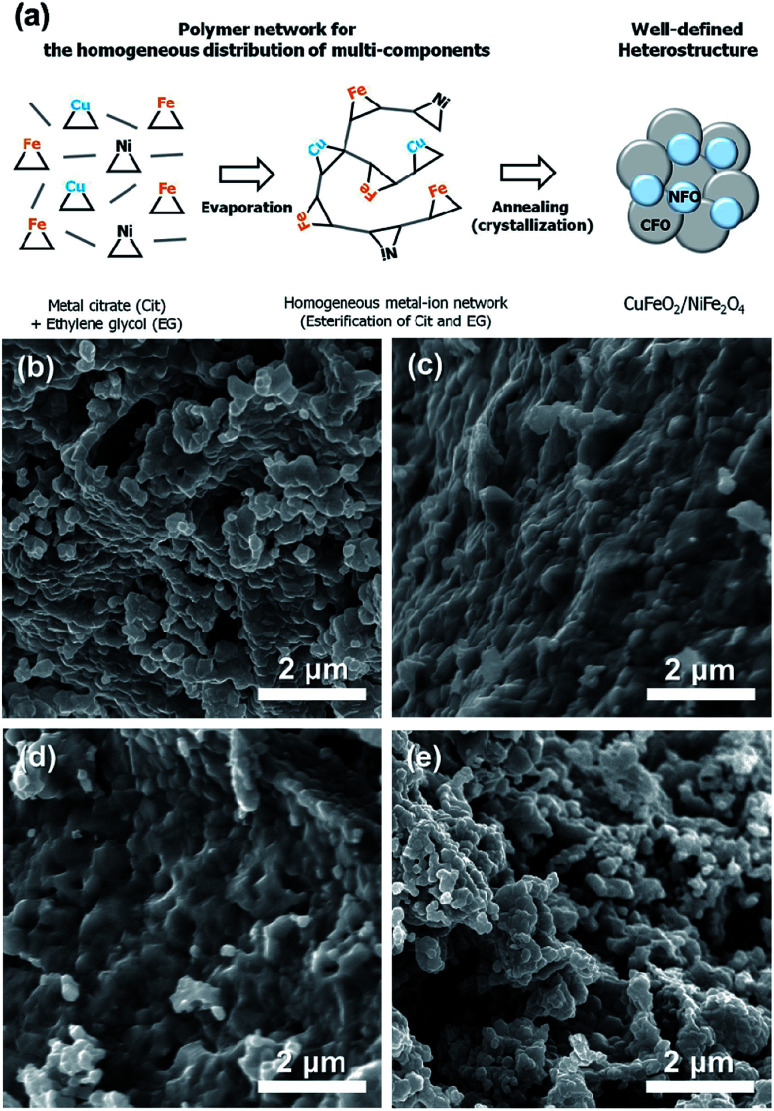
(a) Schematic illustration on the synthesis of CFO and CFO-Ni samples. SEM images of (b) CFO, (c) CFO-Ni (0.1), (d) CFO-Ni (0.2), and (e) CFO-Ni (0.4).

Schematic illustration on the synthesis of CuFeO_2_-Ni composites is presented in Fig. 1a. Initially, Cu, Fe, and Ni precursor were dissolved together in a solution, which forms an interconnected network of metal ions. Upon heat treatment, these interconnected networks are crystallized into delafossites (CuFeO_2_) and spinel (NiFe_2_O_4_), forming heterostructures. The addition of Ni precursor is critical to the synthesis of NiFe_2_O_4_-Ni ions can be easily incorporated into the spinel structure in the presence of Fe and O, where the careful modulation in the amount of Ni precursor is required. For the synthesis of pristine CuFeO_2_, all the procedures were identical except the input of Ni precursor, and the interconnected metal ion network was also formed and later crystallized in a similar manner. To further delve into morphological properties, SEM images of CFO, CFO-Ni (0.1), CFO-Ni (0.2), and CFO-Ni (0.4) were presented (Fig. 1b–e). All of the composites showed formation of nanograins that were connected with one another. The morphologies did not significantly alter due to the introduction of Ni precursor – nevertheless, the elemental composition of CFO and CFO-Ni (0.4) was clearly different, which can be seen by the SEM-EDS analysis of CFO and CFO-Ni (0.4) (Fig. S1†).

**Fig. 2:**
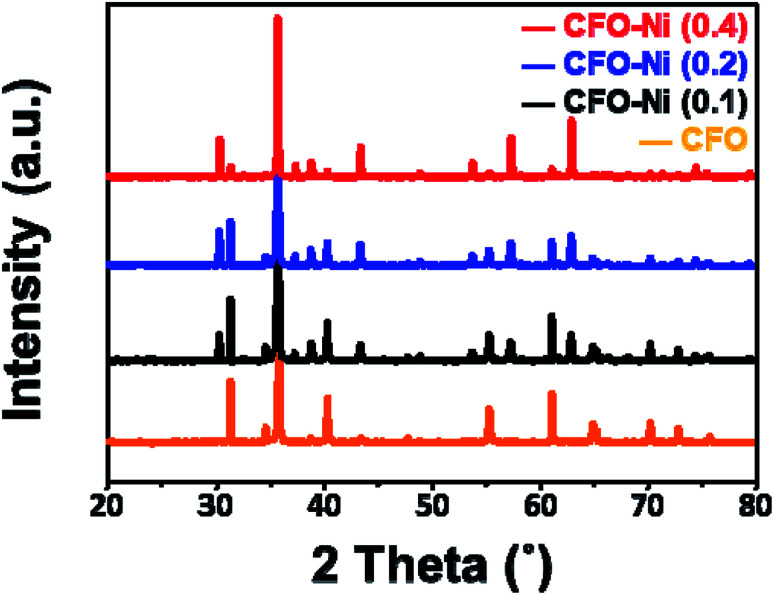
XRD patterns of CFO (orange), CFO-Ni (0.1) (black), CFO-Ni (0.2) (blue), and CFO-Ni (0.4) (red).

To further understand the overall crystal structure of bulk materials, XRD patterns of CFO, CFO-Ni (0.1), CFO-Ni (0.2), and CFO-Ni (0.4) samples were analyzed (Fig. 2). The CFO sample exhibits a delafossite structure, with major crystal planes of (006), (012), (104), (018), and (110), in accordance with JCPDS 75-2146. The addition of Ni precursor induced additional NiFe_2_O_4_ structure (JCPDS 54-0964), as evidenced by additional crystal planes of (220) and (400) at 30° and 43°. The peak intensity of NiFe_2_O_4_ increases as the loading amount of Ni precursor in the sol gel process increases. If the loading amount of Ni precursor is further increased, it is expected that the composite mainly consists of NiFe_2_O_4_, which is not applicable to this study that investigates on the overall properties of CuFeO_2_–NiFe_2_O_4_ hybrid structures.

To explain more on the function of NiFe_2_O_4_ and how they are combined with CuFeO_2_, XPS analysis was carried out (Fig. S2†). The peak of Cu^+^ can be clearly seen at 931.93 eV, and it indicates distinct distributions of two Cu valence states; that is, the signals observed at 931.93 and 933.72 eV correspond to Cu^+^ and Cu^2+^ respectively.^22^ The deconvoluted Cu 2p spectrum shows dominant doublet peaks Cu 2p_3/2_ and Cu 2p_1/2_, corresponding to the binding energies of 931.93 and 951.80 eV with a peak splitting difference of ∼20 eV, indicative of monovalent copper Cu^+^.^22^ In addition, the binding energy (BE) separation (∼13 eV) of photoelectron peaks for Fe 2p_3/2_ and Fe 2p_1/2_, located respectively at 710.73 and 723.94 eV, confirms the Fe^3+^ state of iron.^23^ The satellite peaks observed at 718.99 and 733.13 eV are characteristics of 3+ oxidation state of Fe.^23^ The XPS spectra of Ni show satellite peaks that appear on the high binding energy side of both 2p_3/2_ and 2p_1/2_ regions. It indicates that Ni is in an oxidation state of 2+. The de-convolution of the XPS Ni 2p_3/2_ peak region reveals the presence of two nonequivalent bonds due to two types of lattice sites, tetrahedral and octahedral.^24^ The binding energies associated with Ni 2p_3/2_ is 854.61 and 855.61 eV.^24^

**Fig. 3:**
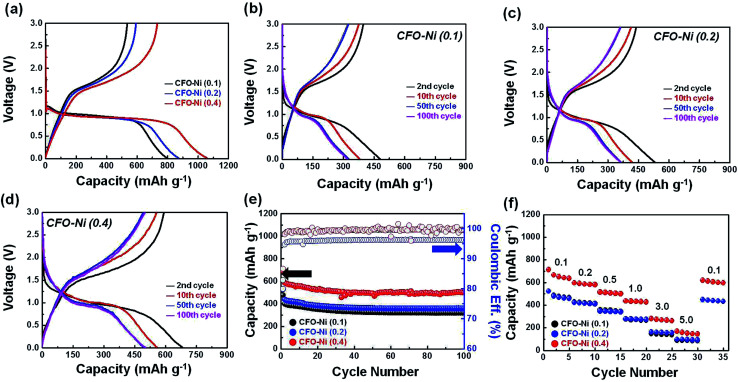
(a) Charge and discharge profile of CFO-Ni (0.1), CFO-Ni (0.2), and CFO-Ni (0.4) in the formation cycle. Charge and discharge profile of (b) CFO-Ni (0.1), (c) CFO-Ni (0.2), and (d) CFO-Ni (0.4) in the 2nd, 10th, 50th, and 100th cycle. (e) Cycle retention characteristics of CFO-Ni (0.1), CFO-Ni (0.2), and CFO-Ni (0.4) at a current density of 500 mA g^−1^ (f) rate capabilities of CFO-Ni (0.1), CFO-Ni (0.2), and CFO-Ni (0.4) at different current densities (expressed in A g^−1^).

The electrochemical performances of CFO-Ni samples were measured by assembling a coin cell, where various CFO-Ni samples were used as working electrodes and Li metal foil as the counter electrode. To understand the irreversible capacity loss in the initial cycle, a voltage profile of CFO-Ni (0.1), CFO-Ni (0.2), and CFO-Ni (0.4) in the formation cycle (50 mA g^−1^) was presented (Fig. 3a). An initial coulombic efficiency (I.C.E.), marked by the ratio between the charge and discharge capacity, was calculated as 67.0, 65.6, and 69.1%, where CFO-Ni (0.4) showed the most reversible reaction with Li. Although not exactly in the same pattern, the introduction of Ni eventually led to higher I.C.E., which can be marked by the catalytic activities that Ni possesses to decompose Li_2_O that was initially formed as a result of conversion reaction.^25^ Additionally, the introduction of Ni resulted in higher charge and discharge capacity in the initial cycle, where CFO-Ni (0.4) delivered a reversible capacity of 740.1 mA h g^−1^, whereas CFO-Ni (0.1) and CFO-Ni (0.2) delivered only 534.8 and 572.9 mA h g^−1^ at a current density of 50 mA g^−1^. During the discharge process, all three samples had a plateau at 0.95 V, which can be assigned to the two-phase reaction that takes place as a result of conversion reaction, in accordance with the previous work.^19^ No noticeable plateau corresponding to the conversion reaction of NiFe_2_O_4_ is shown as it undergoes conversion reaction at a similar voltage.^26^

To compare the trends in redox reactions with Li, the charge and discharge profile of CFO-Ni (0.1), CFO-Ni (0.2), and CFO-Ni (0.4) in the 2nd, 10th, 50th, and 100th cycle is further presented (Fig. 3b–d). It is important to highlight that all the samples show similar voltage profiles, although their reversible capacity and the degree of capacity fading are largely different. The charge and discharge profile of CFO-Ni (0.1) is presented in Fig. 3b, where the capacity faded below 320 mA h g^−1^ after 100th cycle. Nevertheless, the voltage plateau was maintained at 0.95 V even after the 50th and 100th cycle. Secondly, the charge and discharge profile of CFO-Ni (0.2) is presented (Fig. 3c). Compared with CFO-Ni (0.1), it exhibits slightly improved electrochemical performance, where the reversible capacity was maintained to 348.6 mA h g^−1^. The voltage profiles were generally similar between CFO-Ni (0.1) and CFO-Ni (0.2). However, when the concentration of Ni increased, the electrochemical performance was largely enhanced. For CFO-Ni (0.4), the reversible capacity was maintained about 500 mA h g^−1^ even after 100 cycles, while the voltage profiles did not change significantly. Upon larger loading amount of Ni, which forms NiFe_2_O_4_, the overall capacity was significantly enhanced. Nevertheless, without the presence of CuFeO_2_, the pristine NiFe_2_O_4_ alone cannot sustain the good reversible capacity, in accordance with the previous literature.^27^ Through this study, it can be suggested that the considerable loading amount of NiFe_2_O_4_ with minimal amount of CuFeO_2_ is desirable to achieve enhanced reversible capacity with good cycle retention characteristics.

The cycle retention tests in terms of capacity (mA h g^−1^) and coulombic efficiency (%) were further presented to clearly investigate the difference in electrochemical performance of three respective CFO-Ni samples (Fig. 3e). As evidenced by the charge and discharge profiles above (Fig. 3b–d), CFO-Ni (0.4) showed extremely outstanding electrochemical performance, compared with two other CFO-Ni samples (CFO-Ni (0.1) and CFO-Ni (0.2)). CFO-Ni (0.4) exhibits stable electrochemical performance up to 100 cycles at a current density of 500 mA g^−1^, with good reversibility. CFO-Ni (0.2) exhibits slightly enhanced electrochemical performance compared with CFO-Ni (0.1), but shows less coulombic efficiency, which means that its redox reaction with Li is not reversible. Finally, rate capabilities tests (Fig. 3f) were conducted to examine the electrochemical performance of CFO-Ni samples at different current densities (expressed in A g^−1^). Just like in the cycle retention tests (Fig. 3e), CFO-Ni (0.4) exhibits superior electrochemical performance at all current densities (from 0.1 to 5.0 A g^−1^), compared with CFO-Ni (0.1) and CFO-Ni (0.2). Even at a current density of 5000 mA g^−1^, CFO-Ni (0.4) has an average reversible capacity above 150 mA h g^−1^, whereas other two CFO-Ni samples showed a capacity below 100 mA h g^−1^ at the given current density. The capacity of CFO-Ni (0.4) also relapses well to >600 mA h g^−1^ when the current density was changed from 5000 to 100 mA g^−1^.

**Fig. 4:**
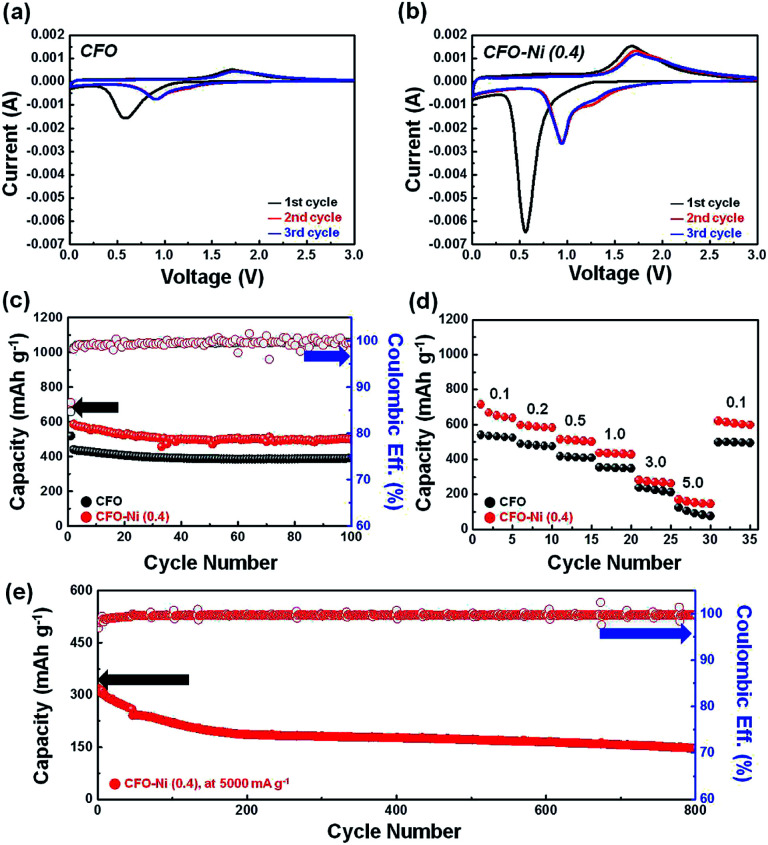
CV curves of (a) CFO and (b) CFO-Ni (0.4). Comparison of electrochemical performance in terms of (c) cycle retention characteristics (at 500 mA g^−1^) and (d) rate capabilities (expressed in A g^−1^) for CFO and CFO-Ni (0.4). (e) High-rate cyclability tests of CFO-Ni (0.4) at a ultra-high current density (5000 mA g^−1^) for 800 cycles.

To clearly compare the effect of Ni introduction in enhancing the electrochemical performances, the electrochemical properties and performances of CFO-Ni (0.4) were compared with pristine CFO synthesized by the same procedure. To probe into the redox reactions with Li, cyclic voltammetry (CV) analysis was conducted for both pristine CFO (Fig. 4a) and CFO-Ni (0.4) (Fig. 4b). In the cathodic scan in the 1st cycle for CV curve of CFO (Fig. 4a), the one major broad peak appears at 0.6 V, which can be ascribed to the conversion reaction of CuFeO_2_ (eqn (1)), which can be written as below:^19^

1 CuFeO_2_ + 4Li^+^ + 4e^−^ → Cu^0^ + Fe^0^ + 2Li_2_O

In the anodic scan in the 1st cycle, one major peak at 1.7 V is ascribed to the oxidation of Cu^0^ and Fe^0^ (eqn (2) and (3)), which can be written below:^19^

2 2Cu^0^ + Li_2_O → Cu_2_O + 2Li^+^ + 2e^−^

3 2Fe^0^ + 3Li_2_O → Fe_2_O_3_ + 6Li^+^ + 6e^−^

In the 2nd and 3rd cycle, the major peak in the cathodic scan was shifted from 0.6 to 1.0 V, where conversion reaction continued to take place. Based on the previous literature,^28^ such shift in the major CV peaks in the cathodic scan suggests some degree of irreversibility, which is apparent in many different kinds of metal oxides.^18,21,29^ Nevertheless, in the anodic scan, such shift in the major peaks is not apparent, where the reversible charge reaction took place from the 1st cycle. Surprisingly, based on the CV curves of CFO-Ni (0.4) (Fig. 4b), the overall redox reactions with Li are not significantly different. Although the introduction of Ni resulted in the formation of new phase (NiFe_2_O_4_), the major peak in the cathodic scan was at 0.6 V, similar to CFO. This is attributed to the fact that the conversion of NiFe_2_O_4_ (eqn (4)) also takes place in the similar voltage. According to the previous literature,^30^ the major cathodic peak in the 1st cycle was located at around 0.54 V, near the voltage peak at which cathodic peak for CFO was also located. Similarly, anodic peak at 1.7 V was present for CFO-Ni (0.4) in the 1st, 2nd, and 3rd cycle, which can be ascribed to the oxidation of not only Cu^0^ and Fe^0^ but also Ni^0^, where additional oxidation of Fe^0^ and Ni^0^ takes place as a result of the formation of NiFe_2_O_4_ (eqn (5)), as shown below:

4 NiFe_2_O_4_ + 8Li^+^ + 8e^−^ → 2Fe^0^ + Ni^0^ + 4Li_2_O

5 Ni^0^ + 2Fe^0^ + 4Li_2_O → Fe_2_O_3_ + NiO + 8Li^+^ + 8e^−^

Based on the comparison of CV curves for CFO and CFO-Ni (0.4), it is clear that the introduction of Ni to form a spinel structure (NiFe_2_O_4_) does not result in significantly different voltage profiles and/or redox reaction mechanisms with Li. This also highlights the synergistic effects that Ni atom can bring together with Fe and Cu.

To better compare the electrochemical performance of CFO and CFO-Ni (0.4), the cycle retention characteristics (Fig. 4c) and rate capabilities (Fig. 4d) were further compared for both samples. Before comparison, the charge and discharge profile of CFO in the formation cycle was presented (Fig. S3†), where the I.C.E. was calculated as 69.6%. Contrary to what was expected, introduction of Ni did not significantly contribute to the higher reversible reaction with Li. Nevertheless, in terms of cycle retention, a clear difference was extant. The charge and discharge profile of CFO in the 2nd, 10th, 50th, and 100th cycle is also shown in Fig. S4,† where it shows significantly lower reversible capacity compared with CFO-Ni (0.4). At a current density of 500 mA g^−1^, CFO-Ni (0.4) exhibits a reversible capacity of ∼500 mA h g^−1^ after 100 cycles, whereas pristine CFO exhibits a reversible capacity of ∼390 mA h g^−1^ after cycling. Similarly, introduction of Ni also resulted in superior rate capabilities (Fig. 4d). At high current densities (3000 and 5000 mA g^−1^), lower reversible capacity is observed for CFO, which shows the limited electrochemical performance of CFO under the condition where fast electron transport is required. Lastly, to investigate the high-rate cyclability, the cycle retention characteristics of CFO-Ni (0.4) were tested (Fig. 4e) at a current density of 5000 mA g^−1^. CFO-Ni (0.4) maintains a reversible capacity of 147.1 mA h g^−1^ at a current density of 5000 mA g^−1^ even after 800 cycles, with an excellent coulombic efficiency of 99.8%. The initial capacity fading in the initial 100 cycles can be related to limited capacity arising from the diffusion barrier that stems from the fast electron transport, where CFO-Ni (0.4) possesses a capacity in the range of 150–160 mA h g^−1^ based on the rate capabilities tests at the identical current density (5000 mA g^−1^) (referred from Fig. 3f). Eventually, after the 200th cycle, CFO-Ni (0.4) shows very outstanding cycle retention characteristics, which are difficult to achieve for so-far reported CuFeO_2_-based electrode materials. The high-rate cyclability shown in this work far surpasses the electrochemical performance of previously reported literatures (Table S1†), although very simple, feasible approach was adopted to fabricate various CFO-Ni samples.

**Fig. 5:**
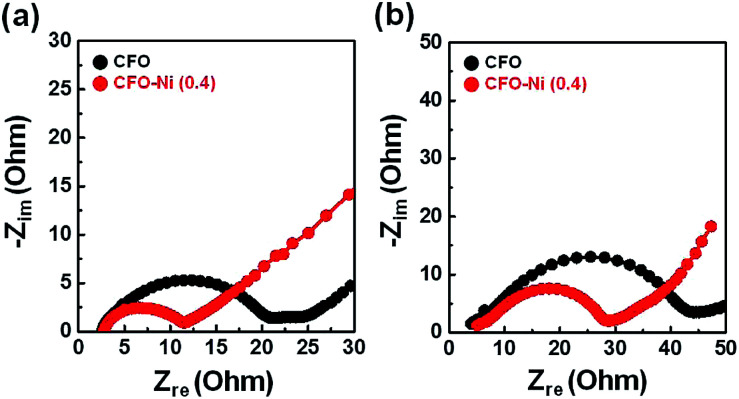
Nyquist plots of CFO and CFO-Ni (0.4) after the (a) 1st cycle and (b) 100th cycle.

A number of parameters need to be considered to delve into the reasons for improved electrochemical performance of CFO-Ni (0.4). To account for the electronic conductivity of electrodes, impedance tests were further conducted for CFO-Ni (0.4) and CFO after the 1st cycle (Fig. 5a) and 100th cycle (Fig. 5b). In both cases, the charge transfer resistance (*R*_CT_) of CFO-Ni (0.4) was significantly smaller than that of CFO, which can be attributed to the introduction of Ni precursor that forms NiFe_2_O_4_ that renders additional electron pathway. *R*_CT_ of both CFO-Ni (0.4) and CFO increases after the 100th cycle, which is apparent due to partial agglomeration that takes place for both samples. Based on impedance tests, formation of heterostructure with NiFe_2_O_4_ resulted in smaller internal cell resistance, which led to enhanced electrochemical performance of CFO-Ni (0.4).

**Fig. 6:**
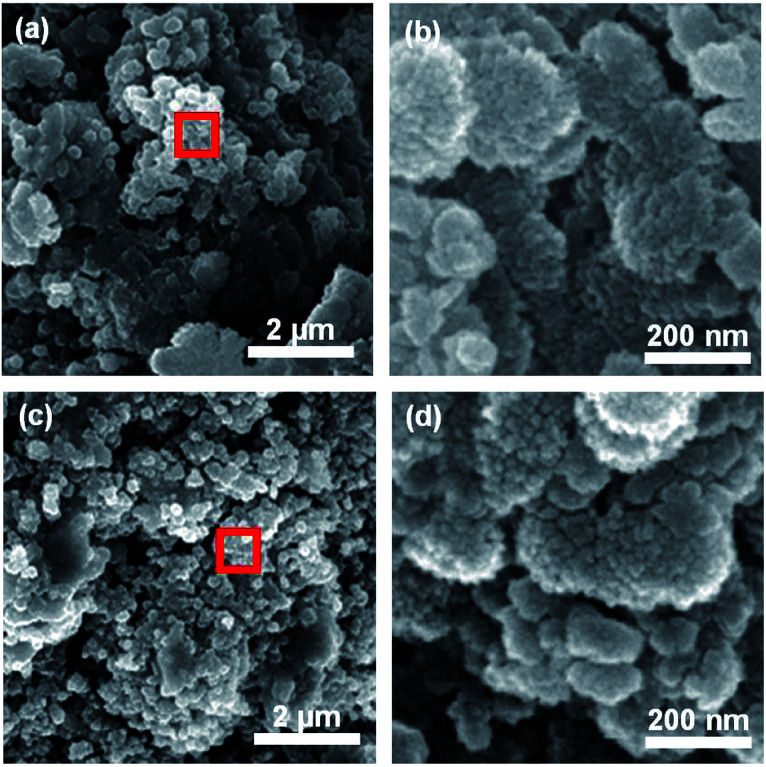
(a) *Ex situ* SEM images and (b) magnified image of red box in (a) for CFO-Ni (0.4) and (c) *ex situ* SEM images and (d) magnified image of red box in (c) for CFO after 100th cycle.

To further compare the overall morphologies, *ex situ* SEM analyses of CFO and CFO-Ni (0.4) after 100 cycles were further compared (Fig. 6). Overall, both CFO and CFO-Ni still maintained their structural integrity, which account for the stable electrochemical performance shown in Fig. 4. Nevertheless, in terms of microstructures, slight difference can be observed. When both CFO-Ni (0.4) and CFO were viewed in high magnification (Fig. 6b and d), the results suggest that less degree of pulverization takes place for CFO-Ni (0.4), which can be attributed to the formation of heterostructures between NiFe_2_O_4_ and CuFeO_2_. When viewed in low magnification (Fig. 6a and c), similar trends were observed, where pulverization took place more actively for CFO, compared with CFO-Ni (0.4). This accounts for decreased *R*_CT_ for CFO-Ni (0.4) compared with that for CFO (Fig. 5) – although both CFO-Ni (0.4) and CFO exhibit similar I.C.E. (∼69%), less degree of pulverization takes place for CFO-Ni (0.4) as both CuFeO_2_ and NiFe_2_O_4_ continuously prevent each other from pulverization. To understand the effect of crystallinity, *ex situ* XRD patterns were carried out (Fig. S5†). Both CFO and CFO-Ni (0.4) exhibit amorphous state, where the amorphization process took place after cycling. Based on these results, introduction of Ni not only brought stable redox reactions with Li, but also minimized cell resistance as well as prevented further pulverization of electrode materials.

## Conclusions

We have successfully synthesized hybrid electrode (Ni–CFO) by simple sol–gel process and subsequent heat treatments, demonstrating outstanding electrochemical performance. Through the introduction of Ni precursor, CuFeO_2_–NiFe_2_O_4_ heterostructure was fabricated, where NiFe_2_O_4_ is expected to improve the low theoretical capacity of CuFeO_2_. Various CFO-Ni samples with different concentration of Ni precursor were fabricated, and CFO-Ni (0.4) in particular exhibited highly reversible reaction with Li, stable high-rate cyclability, and improved rate capabilities. Such outstanding electrochemical performance can be attributed to minimal cell resistance by introducing Ni precursor, and less degree of pulverization upon the synthesis of heterostructures. This work paves a milestone for easily synthesizing heterostructure using sol–gel process and subsequent heat treatments, which is expected to be extended various electrode materials for alternative energy storage system.

## Conflicts of interest

There are no conflicts to declare.

## Supplementary Material

RA-009-C9RA03187A-s001
