# Supplementary material for: CuFeO2–NiFe2O4 hybrid electrode for lithium-ion batteries with ultra-stable electrochemical performance
Source: RSC Adv. 2019 Sep 2;9(47):27257–63. doi: 10.1039/c9ra03187a (PMC9070757; doi:10.1039/c9ra03187a)
Supplement: RA-009-C9RA03187A-s001 [file RA-009-C9RA03187A-s001.pdf]

Electronic Supplementary Material (ESI) for RSC Advances.
This journal is © The Royal Society of Chemistry 2019

$CuFeO_2$-$NiFe_2O_4$ hybrid electrode for lithium-ion batteries with ultra-stable electrochemical performance

Jun Young Cheong [a], Seokwon Lee [b], Jiyoung Lee [a], Haeseong Lim[a], Su-Ho Cho [a], Doh C. Lee [b] and Il-Doo Kim *[a]

[a] Department of Materials Science & Engineering, Korea Advanced Institute of Science & Technology, 335 Science Road, Daejeon, 305-701, Republic of Korea

[b] Department of Chemical and Biomolecular Engineering, Korea Advanced Institute of Science & Technology, 335 Science Road, Daejeon, 305-701, Republic of Korea

*Email address: idkim@kaist.ac.kr (I. D. Kim)

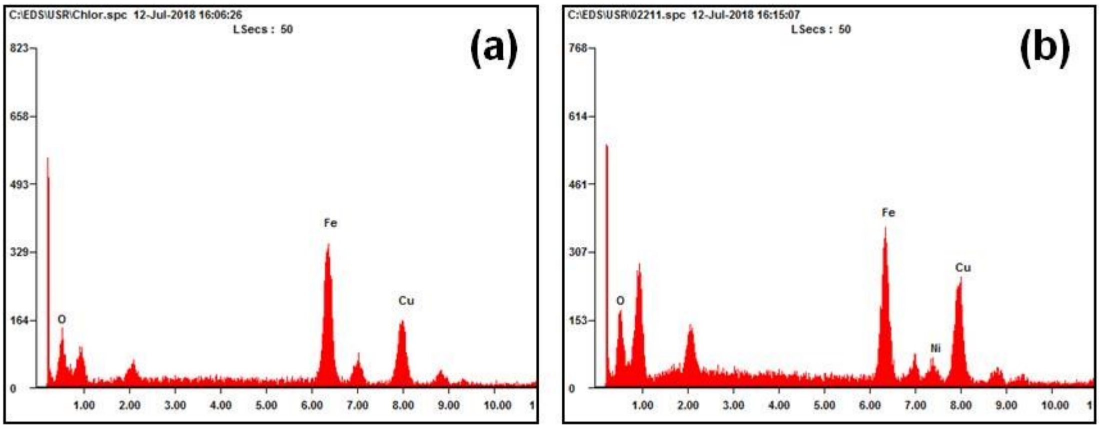

Fig. S1. SEM-EDS analysis of (a) CFO and (b) CFO-Ni (0.4).

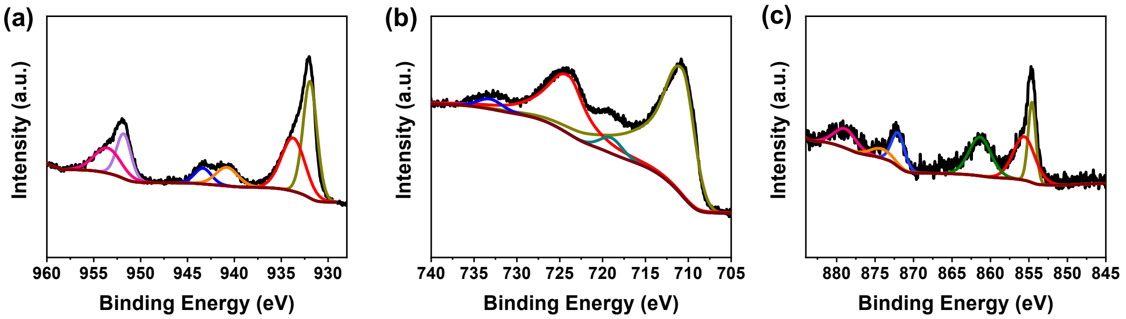

Fig. S2. XPS spectrum for (a) Cu, (b) Fe, and (c) Ni for CFO-Ni (0.4).

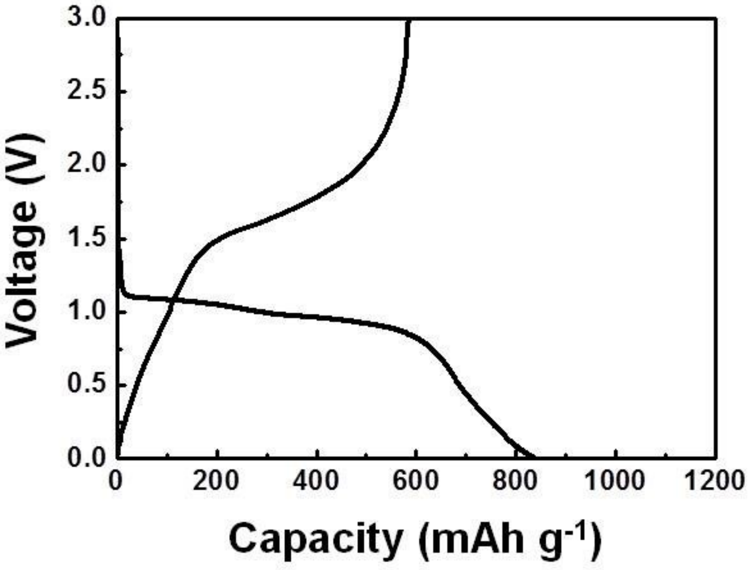

Fig. S3. Charge and discharge profile of CFO in the formation cycle.

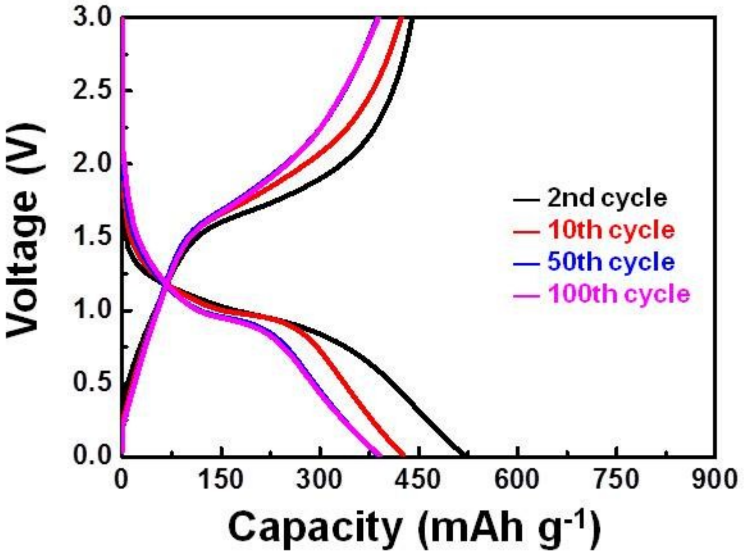

Fig. S4. Charge and discharge profile of CFO in the 2nd, 10th, 50th, and 100th cycle.

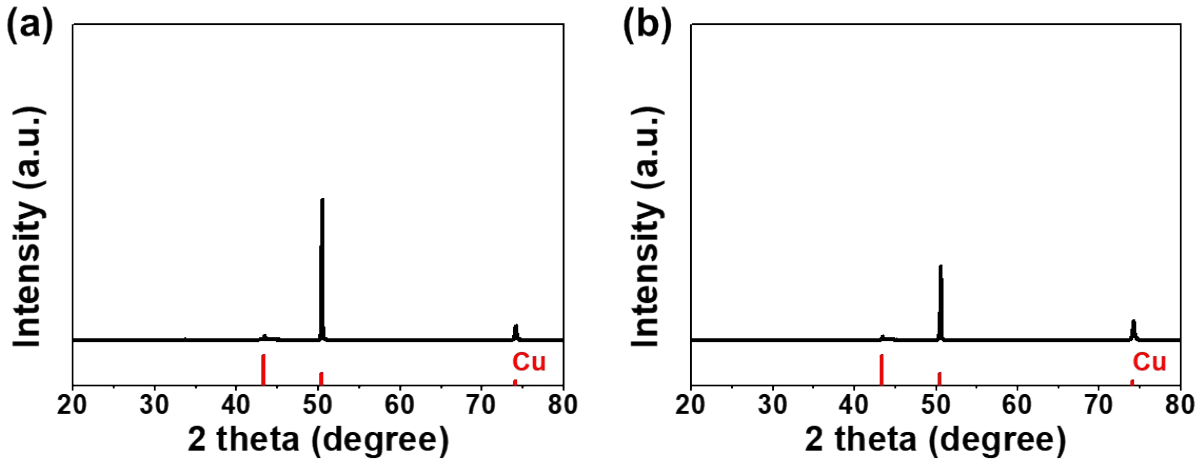

Fig. S5. *Ex situ* XRD patterns of (a) CFO and (b) CFO-Ni (0.4) after cycling.

Table S1. Comparison of electrochemical performance for Co_3O_4 NPs by fast formation cycling with previously reported Co_3O_4-based electrodes.

Sample	Capacity (mAh g^{-1})	Current Density (mA g^{-1})	Cycles	References
$CuFeO_2$@rGO	587	200	100	[1]
$CuFeO_2$/graphene	670	141.6	100	[2]
$CuFeO_2$ (650 ˚C)	475	354	100	[3]
**CFO-Ni (0.4)**	**147**	**5000**	**800**	**This Work**
**CFO-Ni (0.4)**	**500**	**500**	**100**	**This Work**

References

[1] J. Wang, Q. Deng, M. Li, K. Jiang, J. Zhang, Z. Hu, J. Chu, Copper ferrites@reduced graphene oxide anode materials for advanced lithium storage applications, Sci. Rep. 7 (2017) 8903.

[2] Y. Dong, C. Cao, Y.-S. Chui, J.A. Zapien, Facile hydrothermal synthesis of $CuFeO_2$ hexagonal platelets/rings and graphene composites as anode materials for lithium ion batteries, Chem. Commun. 50 (2014) 10151-10154.

[3] L. Lu, J.-Z. Wang, X.-B. Zhu, X.-W. Gao, H.-K. Liu, High capacity and high rate capability of nanostructured $CuFeO_2$ anode materials for lithium-ion batteries, J. Power Sources 196 (2011) 7025-7029.
